# Effects of a brief Snacktivity-based educational intervention on physical activity and exercise behavior among university students

**DOI:** 10.3389/fpubh.2026.1885563

**Published:** 2026-07-24

**Authors:** Zeliha Çelik, Zeynep Pelin Dündar

**Affiliations:** 1 Department of Physiotherapy and Rehabilitation, Amasya University Faculty of Health Science, Amasya, Türkiye; 2 Department of Physiotherapy and Rehabilitation, Nezahat Kelesoglu Faculty of Health Sciences, Necmettin Erbakan University, Konya, Türkiye

**Keywords:** behavior, exercise, physical activity, sedentary behavior, Snacktivity

## Abstract

**Background:**

Snacktivity approach helps reduce sedentary behavior, increase awareness of opportunities for movement, and provide a practical alternative for individuals experiencing time-related barriers to exercise participation through short, integrated physical activity sessions in daily life. However, the effects of short-term Snacktivity-based interventions on university students remain unclear. Therefore, this study aimed to investigate the effects of a brief Snacktivity-based training intervention on physical activity levels, exercise self-efficacy, processes of exercise behavior change, and stages of change in university students.

**Methods:**

This controlled study included 196 undergraduate students in the final analysis (Snacktivity group, *n* = 98; control group, *n* = 98). The intervention group participated in a single Snacktivity educational session and received a brochure containing practical examples of daily activity snacks. Physical activity levels were assessed using the International Physical Activity Questionnaire-Short Form (IPAQ-SF). Exercise Processes of Change Scale (EPCS), Exercise Self-Efficacy Scale, and Exercise Stages of Change Scale were used to evaluate behavioral and psychological factors related to exercise. Assessments were completed at baseline and after a 2-week follow-up period.

**Results:**

Moderate-intensity physical activity significantly increased, while sitting time significantly decreased in the Snacktivity group following the intervention (*p* = 0.007 and *p* = 0.030, respectively). In addition, counter conditioning scores significantly increased in the Snacktivity group (*p* = 0.033), whereas self re-evaluation and self-liberation scores significantly decreased in the control group (*p* = 0.001 and *p* = 0.034, respectively). Following the intervention, the Snacktivity group showed a slight increase from the preparation stage to the action stage, indicating a tendency toward more regular physical activity behavior. In contrast, the control group showed an increase in the contemplation stage. However, the between-group comparison was not statistically significant (*p* = 0.102). No significant changes were observed in vigorous-intensity physical activity, total physical activity, exercise self-efficacy, or total EPCS scores (*p* > 0.05) in the Snacktivity group.

**Conclusion:**

A brief Snacktivity-based educational intervention may positively influence moderate-intensity physical activity participation, sitting time, and certain behavioral processes associated with exercise behavior change among university students. These findings suggest that even a single-session Snacktivity education may support movement-oriented behavior in university students.

## 1 Introduction

Physical activity is one of the fundamental health behaviors recognized for its role in reducing the risk of cardiovascular diseases, type 2 diabetes, obesity, and premature mortality. The World Health Organization (WHO) 2020 Guidelines on Physical Activity and Sedentary Behavior emphasized the importance of regular physical activity for health and stated that all movement throughout the day may provide health benefits (1). However, physical inactivity and sedentary behavior continue to be major public health problems worldwide. Prolonged sitting behavior has been reported to negatively affect cardiometabolic health, mental health, and quality of life (1). University students are considered a population at risk for insufficient physical activity. Academic workload, exam-related stress, lack of time, prolonged study periods, and increased screen time may contribute to sedentary behavior among students (2–4). Physical activity behavior is a multidimensional construct influenced by a range of individual and environmental factors (5). Recent studies have shown that a considerable proportion of university students do not meet the recommended physical activity levels and exhibit high levels of sedentary behavior (2, 3). Therefore, there is a need for feasible, sustainable, and easily integrated physical activity approaches for university students. In recent years, interest in short-duration physical activity approaches that can be integrated into daily life has increased. In particular, “exercise snacks” or short bouts of physical activity have been reported to have positive effects on health (6). Snacktivity is an innovative approach that aims to increase physical activity through short bouts of movement distributed throughout the day. This approach includes short activity snacks lasting several minutes, such as climbing stairs, taking short walks, breaking up sitting time, or incorporating brief movement breaks during daily activities. The Snacktivity approach suggests that physical activity does not consist solely of structured exercise sessions and that short-duration movements may also provide important health benefits (7). Although physiotherapy and rehabilitation students theoretically understand the health benefits of physical activity due to their professional education, their own physical activity levels may remain below the recommended levels. Previous studies have shown that the Snacktivity approach is perceived as feasible, accessible, and motivating by individuals (8–10). Participants reported that they were able to integrate this approach more easily into their daily lives, that their awareness of opportunities for physical activity increased, and that perceived barriers to physical activity decreased (8, 9). In addition, short bouts of physical activity have been suggested to support physical activity behavior, particularly among individuals with limited time (6). The Transtheoretical Model (TTM) proposes that changes in physical activity behavior occur through progressive changes in cognitive and behavioral processes (11). Counter conditioning refers to replacing sedentary or unhealthy behaviors with healthier alternatives, while self-liberation reflects individuals’ commitment and intention to engage in behavioral change (11). The Snacktivity approach may influence these processes by encouraging individuals to replace prolonged periods of sitting with short bouts of physical activity integrated into their daily routines and by increasing awareness of available movement opportunities (7, 8). Therefore, evaluating these processes of change may provide important insights into the behavioral mechanisms through which a short-term Snacktivity intervention may influence exercise behavior. Recent studies have demonstrated that physical activity interventions may be effective strategies for improving movement behaviors among university students. Web-based, motivational, and behavior change-based interventions have shown potential for increasing physical activity participation and improving psychological determinants of exercise behavior in this population (12–14). Although there is growing interest in physical activity-promoting approaches, little is known about the effects of Snacktivity-based interventions on physical activity levels and behavioral processes among university students. Therefore, the aim of this study was to investigate the effects of a Snacktivity-based educational intervention on physical activity levels, exercise self-efficacy, processes and stages of exercise behavior change among university students. The present study was designed to test the following hypotheses:

*H1*: A brief Snacktivity-based educational intervention would increase physical activity levels and reduce sedentary behavior among university students.

*H2*: A brief Snacktivity-based educational intervention would positively influence exercise behavior change processes by encouraging the replacement of sedentary behaviors with short bouts of physical activity.

*H3*: A brief Snacktivity-based educational intervention would improve exercise self-efficacy and facilitate progression toward more advanced stages of exercise behavior change.

## 2 Methods

### 2.1 Study design and participants

This controlled study was conducted among undergraduate students enrolled in the Department of Physiotherapy and Rehabilitation. Participants who volunteered to participate in the study were allocated to either the Snacktivity intervention group or the control group. A total of 216 students were enrolled and completed the baseline assessment. Due to organizational considerations, students were grouped in clusters of approximately 20 participants for the Snacktivity education sessions. Participants were allocated according to session availability and organizational feasibility. Therefore, group sizes were not completely equal (Snacktivity group, *n* = 109; control group, *n* = 107). Participants were included if they were actively enrolled undergraduate university students, aged 18 years or older, volunteered to participate in the study, had no known chronic disease, and possessed sufficient cognitive ability to understand and respond to the questionnaires. Participants were excluded if they had any medical condition restricting physical activity participation (severe cardiopulmonary disease, neurological disorder, or orthopedic problems) or any cognitive or psychiatric condition that could impair understanding of the educational information or completing the questionnaires. Participants were informed through informational posters displayed at the school. All participants provided informed consent prior to participation. All assessments were completed online using Google Forms. The studies involving humans were approved by Amasya University Non-Interventional Clinical Research Ethics Committee (2025/266). The studies were conducted in accordance with the local legislation and institutional requirements.

### 2.2 Intervention

Participants in the intervention group attended a single 30-min Snacktivity educational session conducted by two researchers. The same standardized presentation was delivered to all participants in the intervention group, ensuring that the duration and content of the session were consistent across educational sessions. The baseline assessment was conducted before the presentation. The educational content included information regarding the Snacktivity approach, benefits of reducing sedentary behavior, and practical examples of integrating short physical activity bouts into daily life. Following the educational session, participants received a brochure prepared according to the Snacktivity literature and containing examples of daily activity snacks. Examples included climbing stairs, walking while talking on the phone, performing sit-to-stand movements while waiting for the kettle to boil, and stepping in place during tooth brushing. The intervention aimed to encourage participants to incorporate short bouts of movement into their daily routines. Snacktivity examples and visual materials were adapted from the figures and resources provided by Sanders et al. (7). No intervention was applied to the control group during the study period. After completion of all follow-up assessments, participants in the control group who requested it were also provided with the same educational session. The second evaluation of all participants was completed 2 weeks later.

### 2.3 Outcome measures

#### 2.3.1 Physical activity levels

Physical activity levels were assessed using the Turkish version of International Physical Activity Questionnaire-Short Form (IPAQ-SF). The questionnaire evaluates walking, moderate-intensity physical activity, vigorous-intensity physical activity, sitting time, and total physical activity level expressed as MET-min/week (15).

#### 2.3.2 Exercise processes of change

Behavioral and cognitive processes associated with exercise behavior change were evaluated using the Turkish version of Exercise Processes of Change Scale (EPCS). The scale assesses cognitive processes (consciousness raising, dramatic relief, environmental re-evaluation, self re-evaluation, and social liberation) and behavioral processes (counter conditioning, helping relationships, reinforcement management, self-liberation, and stimulus control). The scale is a self-report instrument typically rated on a 5-point Likert scale ranging from “never” to “repeatedly,” and higher scores indicate greater use of behavior change strategies related to physical activity adoption and maintenance (11, 16).

#### 2.3.3 Exercise self-efficacy

Exercise self-efficacy was assessed using the Exercise Self-Efficacy Scale. Higher scores indicate greater exercise self-efficacy and stronger confidence in sustaining physical activity across different situations (16).

#### 2.3.4 Stages of exercise behavior change

Participants’ stages of exercise behavior change were evaluated based on the TTM using the Turkish Exercise Stages of Change Scale (ESCS). Individuals were categorized into five stages: precontemplation, contemplation, preparation, action, and maintenance. Individuals who do not intend to begin exercising within the next 6 months are classified in the precontemplation stage. Those who are considering starting physical activity within the next 6 months are categorized in the contemplation stage. Participants who engage in some physical activity but not on a regular basis are placed in the preparation stage. Individuals who have been performing regular exercise for less than 6 months are considered to be in the action stage, while those who have maintained regular exercise for 6 months or longer are classified in the maintenance stage (17).

### 2.4 Statistical analysis

Data analysis was performed using the SPSS statistical software package version 25.0 (IBM Corp., Armonk, NY). Daley et al. (18) reported that a sample size of 80 participants (*n* = 40 per group) was appropriate for a Snacktivity feasibility randomized controlled trial to estimate intervention-related parameters with acceptable precision. Based on this recommendation, the present study aimed to recruit an adequate sample size to evaluate the preliminary effects of a Snacktivity-based educational intervention. Baseline between-group comparisons were performed using the Student’s *t*-test for normally distributed variables and the Mann–Whitney *U* test for non-normally distributed variables, while within-group pre- and post-intervention comparisons were analyzed using the Wilcoxon signed-rank test. Effect sizes were calculated using the formula *r* = *Z*/√*N*, *Z* represents the standardized test statistic and *N* represents the number of observations. Effect sizes were interpreted as small (*r* ≥ 0.10), medium (*r* ≥ 0.30), and large (*r* ≥ 0.50) (19). Descriptive statistics for all variables were presented as both mean ± standard deviation and median (interquartile range, IQR). Categorical variables were analyzed using the chi-square test. A *p*-value of <0.05 was determined statistically significant. The post-hoc power for this study was determined using G*Power software (version 3.1.9.7) (20). Based on the Moderate PA (MET-min/week) values, a post hoc power analysis was conducted using an effect size of 0.236, a significance level of 0.05, and a total sample size of 196 participants. The analysis demonstrated that the study achieved a statistical power of 91% (1 − *β* = 0.91).

## 3 Results

**Figure 1:**
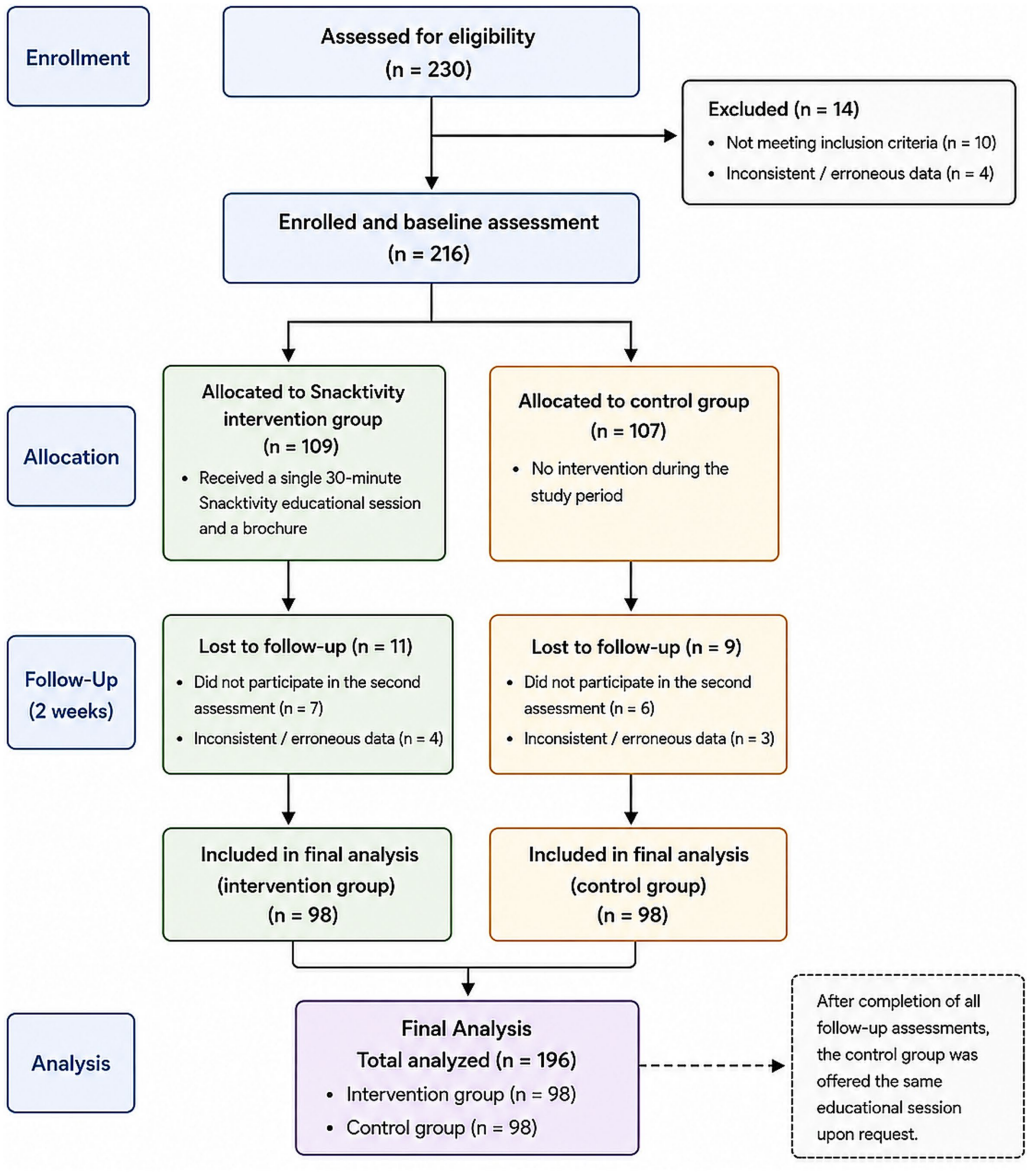
Flow diagram of participant recruitment, allocation, follow-up, and analysis in the intervention and control groups. Participants who did not participate in the second assessment or provided inconsistent/erroneous data were excluded from the final analysis.

A total of 230 students were assessed for eligibility, of whom 216 completed the baseline assessment and were allocated to the Snacktivity group (*n* = 109) or the control group (*n* = 107). During the two-week follow-up period, 11 participants (10.1%) from the Snacktivity group and nine participants (8.4%) from the control group were excluded from the final analysis. In the Snacktivity group, seven participants did not complete the second assessment and four were excluded due to inconsistent data. In the control group, six participants did not complete the second assessment and three were excluded due to inconsistent data. Consequently, 196 participants (98 in each group) were included in the final analysis. The overall attrition rate was 9.3%, and attrition rates were similar between groups (Figure 1). Baseline demographic and clinical characteristics were similar between the groups (Tables 1, 2; *p* > 0.05). Female participants constituted 87.8% of the intervention group and 82.7% of the control group (Table 1).

**Table 1:** Baseline characteristics of participants included in the final analyses.

Characteristics	Snactivity group (*n* = 98; *X* ± SD/median (IQR)	Control group (*n* = 98; *X* ± SD/median (IQR)	*p*
Age, years	21.64 ± 3.73	22.40 ± 2.93	0.116
Female; male, *n*/%	86/87.8%; 12/12.2%	81/82.7%; 17/17.3%	0.314
Weight, kg	64.67 ± 11.71	63.93 ± 11.21	0.650
Height, cm	165.46 ± 8.48	166.64 ± 7.66	0.306
Body mass index, kg/m^2^	23.59 ± 3.75	22.97 ± 3.28	0.215
Smoking exposure (packet × years)	0 (0–0)	0 (0–0)	0.530

kg, kilogram; cm, centimeter; m, meter; BMI, body mass. Between-group comparisons were performed using the Student’s *t*-test for normally distributed variables, while the Mann–Whitney *U* test was used for non-normally distributed variables.

**Table 2:** Changes in variables before and after assessments.

Variables	Snacktivity before	Snacktivity after	Within-group	Control before	Control after	Within-group	Between groups
	*X* ± SD/median (IQR)	*X* ± SD/median (IQR)	p*(*r*)	*X* ± SD/median (IQR)	*X* ± SD/median (IQR)	p*(*r*)	*p* ^b^
Physical activity level							
Moderate PA (MET-min/week)	139.41 ± 280.84 0 (0–240)	245.43 ± 517.75 0 (0–240)	0.007* (0.28)	199.57 ± 440.87 0 (0–240)	306.45 ± 727.20 0 (0–360)	0.326 (0.10)	0.054
Vigorous PA (MET-min/week)	258.42 ± 668.97 0 (0–0)	254.17 ± 644.43 0 (0–0)	0.970 (0.01)	428.63 ± 797.16 0 (0–560)	430.68 ± 873.77 0 (0–480)	0.497 (0.07)	0.058
Total PA (MET-min/week)	1,616.11 ± 1,587.95 1,173 (495–1,986)	1,675.16 ± 1,704.35 1,386 (547.13–2,462.25)	0.317 (0.09)	1,870.85 ± 1,773.91 1,282.25 (671.25–2,452.88)	2,019.54 ± 2,019.27 1,386 (588–2,752.5)	0.817 (0.02)	0.311
Walking (MET-min/week)	1,255.08 ± 1,259.20 907.5 (462–1,386)	1,204.86 ± 1,342.08 693 (404.25–1,386)	0.842(0.02)	1,230.43 ± 1,562.25 693 (396–1,386)	1,311.75 ± 1,573.67 693 (396–1,386)	0.756 (0.03)	0.337
Sitting time (h/day)	7.12 ± 2.22 7 (5.75–9)	6.79 ± 2.61 7 (5–8)	0.030* (0.25)	6.67 ± 2.43 6 (5–8)	5.85 ± 2.78 5 (4–7)	0.063 (0.22)	0.055
EPCS-cognitive processes							
Consciousness raising	13.25 ± 2.83 13 (11–16)	13.03 ± 3.29 13 (11–16)	0.347 (0.10)	14.01 ± 2.95 14 (12–16)	13.42 ± 3.49 14 (11–16)	0.095 (0.17)	0.060
Dramatic relief	12.09 ± 3.38 12 (9–15)	12.41 ± 3.48 12 (10–15)	0.459 (0.08)	12.63 ± 3.37 13 (10–15)	12.18 ± 3.61 12 (10–15)	0.183 (0.13)	0.245
Environmental re-evaluation	12.90 ± 3.28 13 (11–15)	12.88 ± 3.47 13 (10.75–16)	0.877 (0.02)	13.33 ± 3.53 14 (11–16)	13.22 ± 3.61 14 (11–16)	0.845 (0.02)	0.350
Self re-evaluation	13.57 ± 3.27 14 (11–16)	13.49 ± 3.37 14 (12–16)	0.954 (0.01)	14.15 ± 3.38 15 (12–17)	13.23 ± 3.34 14 (11–16)	0.001* (0.32)	0.137
Social liberation	12.41 ± 3.33 12 (10–15)	12.56 ± 3.56 12 (11–15)	0.415 (0.08)	12.42 ± 3.34 13 (10–15)	12.14 ± 3.48 12 (10–14.25)	0.543 (0.06)	0.753
EPCS-behavioral processes							
Counter conditioning	11.46 ± 3.08 12 (9–13)	12.14 ± 3.23 12 (10–14.5)	0.033* (0.22)	12.25 ± 3.27 12 (10–15)	12.43 ± 3.53 12 (10–15.25)	0.838 (0.02)	0.054
Helping relationship	11.91 ± 3.62 12 (9–15)	12.07 ± 3.56 12 (9–15)	0.850 (0.02)	11.92 ± 3.77 12 (9–15)	12.10 ± 3.84 13 (9–16)	0.591 (0.05)	0.933
Reinforcement management	12.91 ± 3.43 13 (11–16)	12.91 ± 3.60 13 (10.75–16)	0.845 (0.02)	13.13 ± 3.42 13 (11–16)	13.24 ± 3.53 13.5 (10.75–16)	0.658 (0.04)	0.630
Self-liberation	14.41 ± 3.77 15 (12–17)	14.20 ± 4.09 16 (12–17)	0.893 (0.01)	14.76 ± 3.51 15 (12–17)	14.07 ± 3.67 15 (12–16)	0.034* (0.21)	0.510
Stimulus control	11.33 ± 3.58 11 (9–13)	12.07 ± 3.54 12 (10–15)	0.085 (0.17)	12.12 ± 3.83 13 (9–15)	12.12 ± 3.94 12 (8–16)	0.828 (0.02)	0.085
EPCS total score (40–200)	125.90 ± 24.80 126 (110.5–144.5)	127.62 ± 29.96 130 (112.5–149.5)	0.504 (0.07)	130.99 ± 28.08 134 (109–151)	128.36 ± 29.99 130 (108–150)	0.391 (0.08)	0.124
Self-efficacy (5–25)	10.38 ± 3.82 10 (7–13)	10.64 ± 3.84 10 (8–13)	0.486 (0.01)	11.00 ± 3.95 10 (8.75–14)	11.46 ± 4.02 11 (10–14)	0.406 (0.08)	0.265

PA, physical activity; MET, metabolic equivalent of task; EPCS, Exercise Processes of Change Scale; IQR, interquartile range; SD, standard deviation; *r*, effect size. Within-group comparisons were performed using the Wilcoxon signed-rank test, while baseline between-group comparisons (*p*^b^) were conducted using the Mann–Whitney *U* test. The symbol * and bold values indicate statistically significant findings (*p* < 0.05).

### 3.1 Physical activity levels

Following the Snacktivity intervention, the Snacktivity group showed a significant increase in moderate-intensity physical activity levels (Table 2; *p* = 0.007) with a small to medium effect size (*r* = 0.28), whereas no significant change was observed in the control group (Table 2; *p* = 0.326). Sitting time was significantly decreased in the Snacktivity group following the intervention (Table 2; *p* = 0.030) with a small to medium effect size (*r* = 0.25), whereas no significant change was found in the control group (Table 2; *p* = 0.063). No significant within-group differences were found for other physical activity levels (Table 2; *p* > 0.05). However, total physical activity scores showed a non-statistical increase in both groups after the follow-up period.

### 3.2 Exercise processes of change

According to cognitive processes of change, no statistically significant differences were found in consciousness raising, dramatic relief, environmental reevaluation, social liberation, or total EPCS scores (Table 2; *p* > 0.05) in the Snacktivity group. However, self re-evaluation scores significantly decreased in the control group (Table 2; *p* = 0.001) with a medium effect size (*r* = 0.32). Among the behavioral processes of change, the Snacktivity group showed a significant increase in counter conditioning scores following the intervention indicating a small effect size (Table 2; *p* = 0.033; *r* = 0.22). The control group demonstrated a significant decrease in self-liberation scores (Table 2; *p* = 0.034) with a small effect size (*r* = 0.21). No significant changes were observed in helping relationships, reinforcement management, or stimulus control scores (Table 2; *p* > 0.05). No statistically significant within-group differences were found in total EPCS scores after the intervention period (Table 2; *p* > 0.05).

### 3.3 Exercise self-efficacy

Exercise self-efficacy scores increased slightly in both groups after the follow-up period. However, these changes were not statistically significant within groups (*p* > 0.05).

### 3.4 Stages of exercise behavior change

**Figure 2:**
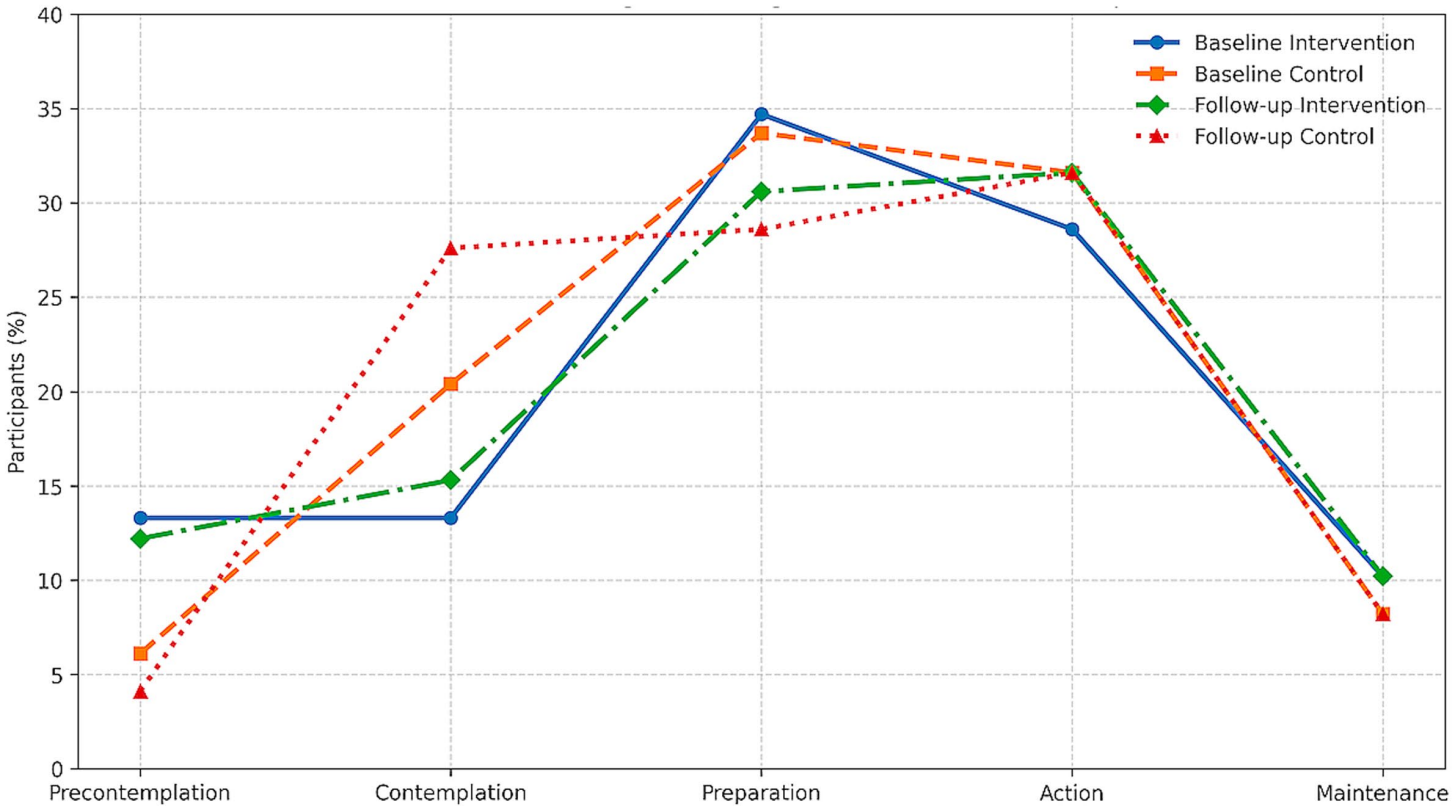
Distribution of stages of behavioral change at baseline and follow-up evaluations in the intervention and control groups. Percentages of participants in precontemplation, contemplation, preparation, action, and maintenance stages are presented for both groups.

At baseline evaluation, in the intervention group, 13 (13.3%) participants were in the precontemplation stage, 13 (13.3%) were in contemplation, 34 (34.7%) were in preparation, 28 (28.6%) were in action, and 10 (10.2%) were in the maintenance stage. In the control group, 6 (6.1%) participants were in precontemplation, 20 (20.4%) were in contemplation, 33 (33.7%) were in preparation, 31 (31.6%) were in action, and 8 (8.2%) were in the maintenance stage (Figure 2, *p* = 0.348).

At follow-up evaluation, in the intervention group, 12 (12.2%) participants were in precontemplation, 15 (15.3%) were in contemplation, 30 (30.6%) were in preparation, 31 (31.6%) were in action, and 10 (10.2%) were in the maintenance stage. In the control group, 4 (4.1%) participants were in precontemplation, 27 (27.6%) were in contemplation, 28 (28.6%) were in preparation, 31 (31.6%) were in action, and 8 (8.2%) were in the maintenance stage (Figure 2, *p* = 0.102).

## 4 Discussion

This study investigated the effects of a single-session Snacktivity-based educational intervention on physical activity levels, exercise behavior processes of change, and exercise self-efficacy among physiotherapy and rehabilitation students. The main findings demonstrated that a short-term Snacktivity education program was associated with increased moderate-intensity physical activity, reduced sitting time, and higher counter-conditioning scores within the intervention group. These findings emphasized that even a brief educational intervention based on the Snacktivity approach may support components of physical activity behavior, particularly moderate-intensity activity participation, sitting time and some behavioral change strategies such as counter-conditioning.

Although no established minimal important difference is available for IPAQ-derived physical activity outcomes in healthy university students, previous research (21) in clinical populations has reported minimal important differences ranging from 13 to 58 min/week for MVPA. The increase observed in the present study (approximately 25–30 min/week of moderate-intensity physical activity) falls within this range. However, because these estimates were derived from clinical populations, direct comparisons with the present findings are limited.

The Snacktivity approach is a relatively new concept that inspires individuals to accumulate physical activity through short, integrated movements in their daily lives. This approach is based on updated physical activity guidelines that emphasize the importance of all movement, regardless of duration (1, 7). In the present study, participants who received a single session of Snacktivity training showed significant improvement in their moderate physical activity levels and sitting times at the end of a 2-week follow-up period. The observed improvement may suggest a potential beneficial association between the Snacktivity intervention and moderate-intensity physical activity participation.

Our results are consistent with previous Snacktivity-related studies reporting that short and manageable activity bouts may reduce perceived barriers to exercise participation. Krouwel et al. (8) reported that individuals perceived Snacktivity as more accessible compared to structured exercise programs and expressed that this approach improved their psychological wellbeing. Tyldesley-Marshall et al. (9) evaluated the opinions of physically inactive adults after they experienced the Snacktivity approach, which involves short “activity snacks” lasting 2–5 min, for 5 days. The findings showed that participants found the Snacktivity approach feasible and motivating, that they became more aware of opportunities for physical activity during the day. Public perception studies also support the acceptability of Snacktivity as a “small changes” approach to reducing sedentary behavior (10). In addition, previous epidemiological, experimental and qualitative studies emphasized that Snacktivity may be particularly beneficial in populations facing time-related barriers (6).

University students around the world are often insufficiently active and highly sedentary. Research consistently identifies time pressures, stress, academic workload, and study-related sitting as main barriers to regular physical activity (2, 3, 22). Therefore, the Snacktivity model may be particularly suitable for this population because it decreases the need for dedicated exercise sessions and instead promotes movement during routine daily activities. However, participant engagement with Snacktivity behaviors was not directly assessed in the present study. In our study, after participants were informed about Snacktivity, they were given a brochure about Snacktivity features and example activities. Corella et al. (12) investigated a behavioral motivation-based intervention among university students in the contemplation stage and reported improvements in physical activity behavior together with positive changes in cognitive variables related to behavior change. In addition, Duan et al. (13) demonstrated that a web-based intervention among Chinese university students improved physical activity behavior and stage progression, although changes across different physical activity intensities were variable. Furthermore, Martens et al. (14) reported that a short-term motivational intervention increased high-intensity physical activity among sedentary university students, while moderate-intensity activity showed weaker and less consistent changes. In contrast to their findings, the present study primarily showed improvement in moderate-intensity physical activity. This may be consistent with the intended focus of the Snacktivity approach, although participant uptake of Snacktivity behaviors was not directly assessed. On the other hand, these findings suggest that the magnitude and pattern of change observed in the present study are broadly consistent with those reported in other brief physical activity interventions conducted among university students (12–14). The brief interventions may influence specific aspects of physical activity behavior, although larger and more sustained effects may require more intensive or longer-term strategies.

Following the intervention, no statistically significant improvement was observed in the vigorous intensity and total physical activity levels of participants in the Snacktivity group. This may be explained by the relatively short intervention duration. Long-term changes in physical activity behavior may depend on repeated reinforcement and continued engagement over time. Studies on health behaviors have emphasized that sustained motivation, self-monitoring, and environmental support are critical for this change to be permanent (23, 24). The single session in our current study may not have been sufficient to quickly change participants’ snacking habits and make the behavioral change permanent.

On the other hand, the participants in this study were physiotherapy and rehabilitation students who may already have a relatively high baseline awareness of the benefits of physical activity. Both groups included individuals with high baseline total physical activity values. This is supported by the exercise behavior stage, which shows that many participants exhibited exercise behavior change during the preparation or action phases of both the baseline and follow-up phases. Consequently, the extent to which the intervention translates into significant behavioral change may be limited.

Findings from the EPCS scale provide additional information regarding physical activity behavior change (11). A significant increase in counter conditioning scores was observed in the intervention group. Counter conditioning refers to the replacement of sedentary or unhealthy behaviors with healthier alternatives and is considered one of the key behavioral processes within the TTM (11). The Snacktivity approach encourages the replacement of sedentary moments with short opportunities for movement (7). Therefore, the increase in counter-conditioning observed in the Snacktivity group is consistent with the theoretical basis of Snacktivity. The observed decreases in self-re-evaluation and self-liberation scores within the control group may indicate changes in psychological processes related to exercise behavior change. However, the underlying reasons for these changes cannot be determined from the present study and may reflect natural temporal fluctuations, testing effects, or other unmeasured factors.

Exercise self-efficacy scores did not improve following the intervention in our study. According to Social Cognitive Theory, sustained engagement and successful behavioral repetition are required to strengthen self-efficacy beliefs. The self-efficacy involves starting new habits, continuing them, overcoming problems, and maintaining those new habits (25, 26). Our current intervention consisted primarily of a brief information session and brochure distribution, without structured follow-up, supervised intervention, or sustained motivational support. Therefore, participants may not have experienced sufficient behavioral reinforcement to meaningfully change their self-efficacy beliefs.

## 5 Limitations

This study had several limitations. First, physical activity levels were assessed exclusively using the self-reported IPAQ-SF questionnaire. Although this instrument is widely used and validated, objective measures such as accelerometers may provide a more comprehensive assessment of physical activity and sedentary behavior. Future studies should therefore consider incorporating objective monitoring methods alongside self-report measures. Second, the intervention consisted of a single 30-min educational session and brochure-based support. The limited intensity and short duration of the intervention may have been insufficient to produce substantial or sustained behavioral changes. Therefore, the findings should be considered preliminary, and future studies with more intensive interventions and longer follow-up periods are needed to confirm these results. In addition, participant adherence to the Snacktivity recommendations was not directly monitored during the follow-up period. Therefore, the extent to which participants implemented Snacktivity behaviors remains unknown, and potential dose–response relationships between intervention uptake and study outcomes could not be examined. Third, the study population consisted of only physiotherapy and rehabilitation students from a single academic setting, which may limit generalizability to other university populations or community samples. Another limitation of this study was the lack of blinding. Neither participants nor researchers were blinded to group allocation, which may have introduced performance and response bias. Furthermore, the wait-list control design may have introduced expectancy-related effects among control participants, which should be considered when interpreting the findings. The multiple outcome analyses were conducted without adjustment for multiple comparisons, which may have increased the risk of type I error. Lastly, participants were not randomly allocated to the intervention and control groups. Group assignment was based on session availability and organizational feasibility, which may have introduced selection bias and limited the ability to establish causal relationships. Although baseline characteristics were similar between groups, unmeasured differences may have influenced the observed outcomes. Therefore, the findings should be interpreted with caution, and future randomized controlled studies are needed to confirm the effectiveness of Snacktivity-based interventions.

## 6 Conclusion

The present study demonstrated that brief Snacktivity education may support moderate-intensity physical activity participation and certain behavioral processes associated with exercise behavior change among physiotherapy students. Although no significant improvements were observed in total physical activity or exercise self-efficacy, the findings suggest that simple, low-burden educational approaches may support movement integration into daily life. Snacktivity may represent a promising approach for promoting physical activity among university students who experience time-related barriers to exercise participation.

## Data Availability

The datasets generated during the current study are not publicly available due to ethical and privacy considerations but are available from the corresponding author upon reasonable request. Requests to access the datasets should be directed to zelihacelik1@hotmail.com.
